# Rap2a serves as a potential prognostic indicator of renal cell carcinoma and promotes its migration and invasion through up-regulating p-Akt

**DOI:** 10.1038/s41598-017-06162-7

**Published:** 2017-07-26

**Authors:** Jin-Xia Wu, Wen-Qi Du, Xiu-Cun Wang, Lu-Lu Wei, Fu-Chun Huo, Yao-Jie Pan, Xiao-Jin Wu, Dong-Sheng Pei

**Affiliations:** 1 Department of Pathology, Xuzhou Medical University, Xuzhou, 221004 China; 2 Department of Physiology, Xuzhou Medical University, Xuzhou, 221004 China; 3 Jiangsu Key Laboratory of Biological Cancer Therapy, Xuzhou Medical University, Xuzhou, 221002 China; 4 Jiangsu Center for the Collaboration and Innovation of Cancer Biotherapy, Xuzhou Medical University, Xuzhou, 221002 China; 5 Department of Neurosurgery, The Affiliated Hospital of Xuzhou Medical University, Xuzhou, 221002 China; 6 Department of Radiation Oncology, The First People’s Hospital of Xuzhou, Xuzhou, 221002 China

## Abstract

Rap2a, a member of the small GTPase superfamily, belongs to Ras superfamily, and its function in cancer progression is still poorly understood. Our previous study indicated that the ectopic expression of Rap2a enhanced the migration and invasion ability of lung cancer cells. However, its expression and molecular mechanism on renal cell carcinoma (RCC) have not been characterized. This study explored the clinical significance and biological function of Rap2a in human RCC. The clinical relevance of Rap2a in RCC was evaluated by immunohistochemical staining using tissue microarray. Our data showed that Rap2a expression was dramatically increased in RCC tissues compared with normal renal tissues. The ectopic expression of Rap2a enhanced the migration and invasive ability of cancer cells. In contrast, downregulation of Rap2a inhibited cell invasion. Rap2a had no effect on the proliferation of RCC cell lines. Meanwhile, Rap2a can regulate the phosphorylation level of Akt *in vitro*. *In vivo* studies also showed that Rap2a positively regulated metastasis of renal cancer cells and the expression of p-Akt. These findings indicate that Rap2a promotes RCC metastasis and may serve as a candidate RCC prognostic marker and a potential therapeutic target.

## Introduction

Kidney cancer is one of the most common malignancies in the world. Around 208,500 new cases of kidney cancer are diagnosed in the world each year, accounting for just under 2% of all cancers^1^. It has been concluded that kidney cancer is fundamentally a metabolic disease and caused by mutations in different genes^2^. Renal cell carcinoma (RCC) is the most common type of kidney cancer in adults. Molecular biomarkers have been shown to aid the diagnosis for several cancers. Thus, a better understanding of the genetic and metabolic basis of RCC may lead to the development of effective forms of therapy for this disease^3^.

Ras-related proteins are composed of a large family of small molecular weight guanine nucleotide binding proteins that are involved in a variety of cellular processes such as proliferation, differentiation, cell adhesion, and cell cycle control^4^. Furthermore, the Rap family has 50–60% sequence homology with the product of the Ras proto-oncogene^5, 6^. In performing their cellular functions, ras-related proteins cycle between inactive GDP-bound and GTP-bound forms^6^. Five different members of this family have been indentified: Rap1a, Rap1b, Rap2a, Rap2b and Rap2c^7, 8^. Rap2a, being one of the members of the Ras superfamily, was predominantly up-regulated in many types of tumors^9, 10^. Previously, we have found that Rap2a is a direct target of p53 and plays an important role in cancer cell migration and invasion. In addition, the ectopic expression of Rap2a is observed in osteosarcoma, and is involved in tumorigenesis through activation of the p-Akt pathway^11^. However, the expression and function of Rap2a have not been fully elucidated in the development of human RCC.

In the current study, we investigated the prognostic significance of Rap2a in the development and progression of RCC. In addition, we constructed RCC cell lines in which Rap2a expression over-expressed or down-regulated to examine the role of Rap2a on the proliferation, migration and invasion of tumor cells. Finally, we investigated the molecular mechanisms by which Rap2a was involved in RCC progression. Our data demonstrated that high expression of Rap2a was significantly associated with RCC occurrence. Meanwhile, Rap2a promoted RCC cells invasion and metastasis by regulating the phosphorylation level of Akt *in vitro* and *in vivo*. These data may indicate that Rap2a is a potential candidate for clinical biomarker, which could be used as a potential prognosis and therapeutic marker for kidney cancer.

## Materials and Methods

### Patients and tissue specimens

The study was conducted in accordance to local ethical guidelines. Renal cell carcinoma tissue microarray (TMA) was purchased from Shanghai Xinchao Biotechnology (Shanghai, China). It included 189 patients who underwent radical nephrectomy from 2006 to 2011. The array dot diameter was 1.5 mm, and each dot represented a tissue spot from one individual specimen that was selected and pathologically confirmed. Tumors were staged according to the 2010 revised TNM system as follows: 127 cases with stages I-II and 62 cases with stages III-IV. The validation cohort TMA consisted of 189 surgical cases and 68 cases of normal renal tissues was constructed by a contract service at the National Engineering Centre for Biochip (Shanghai, China). The studies using human renal tissue samples were approved by Xuzhou Human Subject Committee. Informed consent from the patients was obtained in all cases.

### Immunohistochemistry of TMA

Immunohistochemistry was performed according to the streptavidin-peroxidase (Sp) method using a standard Sp Kit (Zhongshan biotech, Beijing, China). TMA slides were dewaxed and then rehydrated with graded ethanol and distilled water. Endogenous peroxidases were inhibited by 3% H_2_O_2_ and antigen retrieval was performed in a microwave oven with 10 mM citrate buffer (pH 6.0). Then the slide was incubated with monoclonal mouse anti-Rap2a antibody (1:100) overnight and diaminobenzidine was used to produce a brown precipitate. After hematoxylin counterstain and dehydration, the sections were sealed with cover slips. Negative controls were performed by phosphate buffered saline (PBS) replaced Rap2a antibody during the primary antibody incubation. The staining of the normal renal tissues in each microarray slide was evaluated as the quality control of the immunostaining. The immunoreactivity was assessed blindly by two independent observers using light microscopy (Olympus BX-51 light microscope), and the image was collected by Camedia Master C-3040 digital camera.

### Cell lines and growth condition

Renal tubular epithelial cell line HK-2 and human RCC cell lines 786-O, Ketr-3 and ACHN were purchased from the Institute of Biochemistry and Cell Biology, Chinese Academy of Sciences (Shanghai, China). Ketr-3 cells, ACHN cells and HK-2 cells were cultured in DMEM medium supplemented with 10% fetal bovine serum (Invitrogen, Shanghai). 786-O cells were cultured in RPMI1640 medium (Hyclone). Cells were incubated at 37 °C in a humidified atmosphere of 5% CO_2_.

### Plasmid construction

Total RNA was extracted from U2OS cells by using the Qiagen RNeasy kit (Qiagen, USA), and first-strand cDNA was synthesized by the PrimeScript RT reagent kit (Takara) according to the manufacturer’s instructions. Then, the cDNA for Rap2a was amplified using Taq polymerase, and the following primers:

5′-G*AAGCTT*ATGCGCGAGTACAAAG-3′, forward

5′-G*GAATTC*CTATTGTATGTTACATG-3′, reverse.

Consensus sequences for the restriction enzymes ECoRI (in the forward primers) and HindIII (in the reverse primers) are italic.

The cDNA was then subcloned into the bacterial expression vector pcDNA3.1 at ECoRI and HindIII sites. The identity of the resulting clones was verified by sequencing.

### DNA and siRNA transfections, and stable cell line generation

Transfection of pcDNA3.1-control and pcDNA3.1-Rap2a expression the plasmids were carried out using Lipofectamine 2000 transfection reagent (Invitrogen, Shanghai, China) following the manufacturer’s protocol. Rap2a siRNA was purchased from Gene-Pharma (Shanghai, China) and transfected using siLentFect Lipid Reagent (Bio-Rad, Hercules, CA, USA) according to the manufacturer’s instructions. After transfection, cells were harvested for subsequent experiments. The Rap2a overexpression 786-O cell lines and control 786-O cell lines were established by infecting with lentivirus packing Rap2a expression vector (GFP is not fused with Rap2a in this vector) and control vector respectively. Target cells were infected with lentivirus for 48 hours then selected with puromycin (Santa Cruz) for 3 weeks.

### Western blot

24 hours after transfection, cells were harvested from the plates. After digestion and centrifugation, total cell proteins were extracted from the cells using RIPA lysis method. Then, 100 μg proteins were applied onto 12% SDS-PAGE for electrophoresis and then transferred onto nitrocellulose membrane. After blocking for 2 h, members were incubated overnight at 4 °C with the antibodies (Rap2a, 1:1000; Bcl-2, 1:500; Bax, 1:500; Akt, 1:2000; p-Akt, 1:1000; β-actin, 1:2000). After washed, the secondary antibody was added to incubate at room temperature for 1 h prior to ECL fluorescence imaging.

### Cell proliferation assay

786-O, Ketr-3 and ACHN cells were seeded (5 × 10^3^/well) into 96-well culture plates after transfection. Cell proliferation was evaluated by CCK8 (Vicmed, China) at various time points 24 h, 48 h, 72 h and 96 h according to the manufacturer’s instructions. Then, 100 μl serum-free culture medium and 10 μl CCK-8 solution were added into each well, followed by incubation at 37 °C for 1 h. The absorbance was measured at 490 nm using an ELX-800 spectrometer reader (Bio-Tek Inststruments, Winooski, USA). Each experiment was performed in triplicate.

### Detection of apoptosis by flow cytometry with Annexin V-FITC

Cell culture and treatment were performed as described above. The apoptotic rate was detected with Annexin V/PI staining (Nanjing KeyGen Biotech, Nanjing, China) using a fluorescence-activated cell sorting (FACS) machine (FACSCalibur™, Becton-Dicskinson Biosciences, San Diego, CA, USA). The staining procedures were carried out according to the relevant manufacturer’s instructions.

### Wound-healing assay

After transfection with Rap2a plasmid, the RCC cells were cultured. A wound was made by dragging a yellow pipette tip along the centre of the plate. The distance between the cells bordering the wound was measured every 24 h. Images of cells were taken at the time indicated under the phase-contrast microscope with a digital camera.

### Migration assay

A modified two chamber plate with a pore size of 8 μm was used to determine cell migration. 1 × 10^5^ RCC cells were seeded in serum-free medium in the upper chamber. After cultured for 24 h in a 37 °C incubator, the cells were fixed in methanol and stained with leucocrystal violet. Cells in upper chamber were removed with a cotton swab and the number of cells which traversed the membrane was determined by counting the leucocrystal violetstained cells. Stained cells were viewed under a microscope (200× magnification), and the number of migrated cells was counted in the whole field. The assays were performed in triplicate.

### Invasion assay

Transwell inserts with 8 μm-pore-size membranes were coated with 100 μl of diluted (1:8 in serum-free medium) Matrigel (BD Biosciences, NJ, USA) in 24-well plates. 1 × 10^5^ RCC cells were seeded in serum-free medium in the upper chamber. Cells were cultured for 48 h in a 37 °C incubator, and non-invading cells were removed from the upper chamber of each transwell with a cotton swab. Invading cells were fixed in methanol and stained with leucocrystal violet. Stained cells were viewed under a microscope (200× magnification), and the number of migrated cells was counted in the whole field. The assays were performed in triplicate.

### Tail vein metastasis assay

Female BALB/c nude mice were purchased from Beijing HFC Biotechnology (Beijing, China). For tumor model, Rap2a-overexpression 786-O cells and Ctrl-786-O cells were suspended in PBS. The mice were injected intravenously with 2.5 × 10^6^ 786-O cells in 0.2 ml of PBS through tail vein respectively. After 2 months, the two groups of mice were sacrificed, their lungs were resected and fixed in 10% buffered formalin for metastatic nodules counting and further histopathological analysis. The number of metastatic nodules presented on the surface of each set of lungs was counted by visual inspection using a stereoscopic dissecting microscope. The protocols for animal studies were approved by the Institutional Animal Care and Use Committee of Xuzhou Medical University.

### Statistical analysis

For TMA, the association between Rap2a staining and the clinicopathologic parameters of the RCC patients were evaluated by χ2 test. Difference between each patient’s tumor tissues with its normal counterpart was evaluated by χ2 test. All values are shown as means ± SD (standard deviation). Student’s t-tests or one-way analysis of the variance (ANOVA) was performed for determination of *P* values with SPSS 16.0 software. All experiments were performed at least three times unless otherwise indicated. *P* values < 0.05 were considered statistically significant.

## Results

### Rap2a expression is increased in human RCC

**Figure 1:**
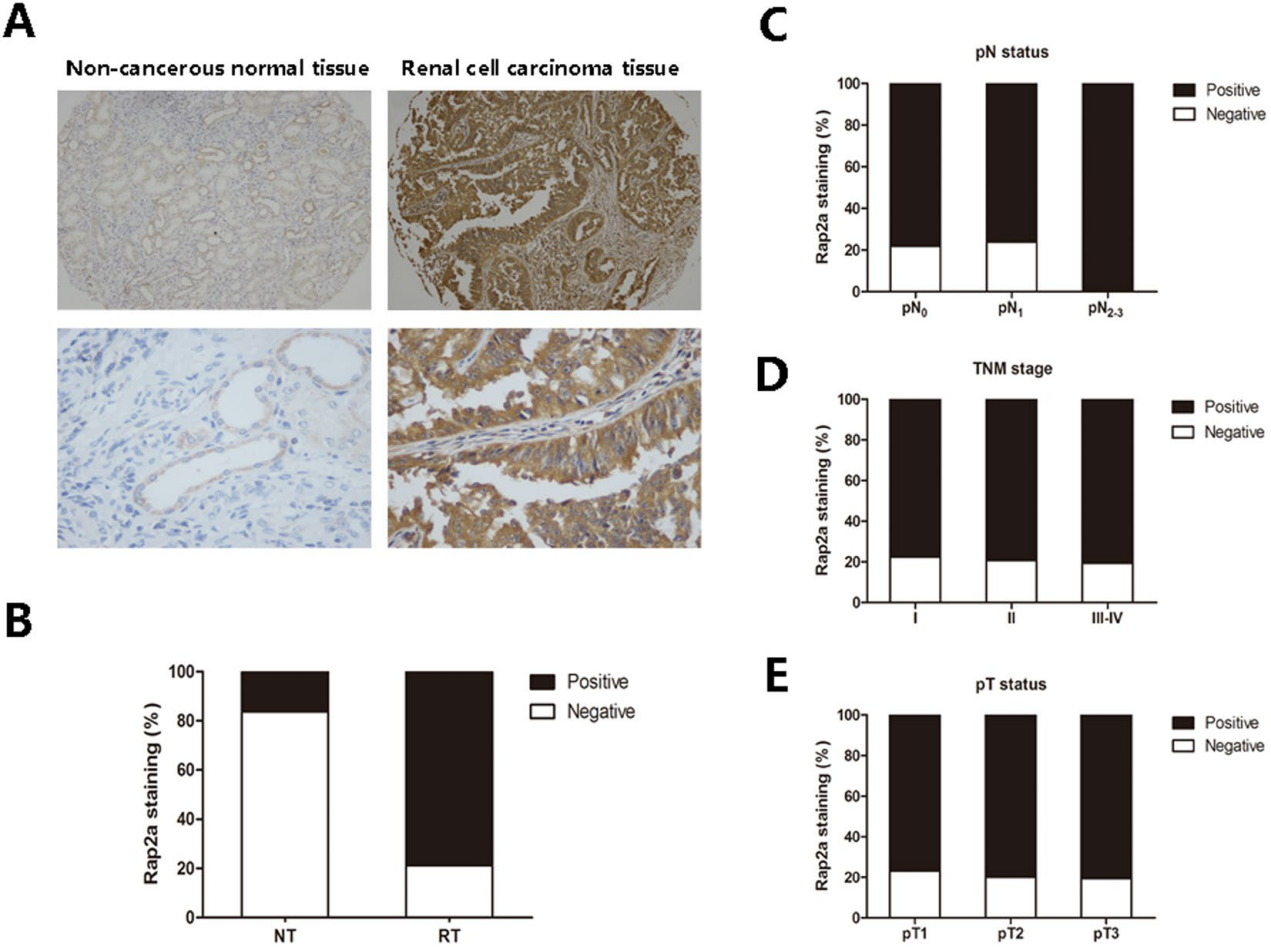
Correlation between Rap2a expression and clinicopathologic parameters in RCC patients. (**A**) Representative photographs showed Rap2a immunohistochemical staining in TMA. They were taken at different magnifications in normal renal tissue and renal carcinoma tissues (Top panel × 100, bottom panel × 400). (**B**) Compared with that in the normal renal tissue, the overall expression level of Rap2a in the renal cell carcinoma tissues was significantly higher (*P* < 0.01, χ^2^ test). (**C**) Increased Rap2a expression was not correlated with lymphatic metastasis. (**D**) Rap2a expression was not correlated with TNM stage. (**E**) Rap2a expression was not correlated with depth of invasion.

To study whether Rap2a expression is changed in human RCC, Immunohistochemistry staining was utilized in TMA slide in normal renal tissues and RCC tissues. We found that Rap2a expression was localized in the cytoplasmic (Fig. 1A). In renal cell carcinoma tissues, there was strong cytoplasmic immunostaining in renal tubule epithelia. Positive Rap2a staining was recorded in 78.8% (149 of 189 cases). Of the 68 patients with non-cancerous normal tissues, positive expression of Rap2a was observed in 16.2% (11 of 68 cases) (Fig. 1B). A significant higher expression of Rap2a was observed in the carcinoma tissues when compared with normal human renal tissues (*P* = 0.000, χ^2^ test).

### Correlation between Rap2a expression and clinicopathological parameters in RCC patients

The relationships between Rap2a expression and clinicopathologic characteristics were summarized in Table 1. We investigated whether Rap2a expression correlated with clinicopathological features, such as depth of invasion-pT status, lymph node metastasis-pN status and TNM stage. However, we did not find significant correlation between Rap2a expression with clinicopathologic variables, including TNM stages (*P* = 0.800), depth of invasion-pT status (*P* = 0.854), lymph node metastasis-pN status (*P* = 0.319) (Fig. 1C–E), age (*P* = 0.518), gender (*P* = 0.375) and tumor size (*P* = 0.516) (Table 1).

**Table 1:** Patients characteristics and Rap2a expression.

Variables	Rap2a staining			
	Negative (%)	Positive (%)	Total	P*
**All cases**	40 (21.2)	149 (78.8)	189	0.000
**Age**				
≤57 years	17 (23.6)	55 (76.4)	72	0.518
>57 years	23 (19.7)	94 (80.3)	117	
**Gender**				
Male	28 (23.1)	93 (76.9)	121	0.375
Female	12 (17.6)	56 (82.4)	68	
**Tumor size**				
≤7 cm	29 (22.5)	100 (77.5)	129	0.516
>7 cm	11 (18.3)	49 (81.7)	60	
**pT status**				
pT_1_	25 (23.1)	83 (76.9)	108	0.854
pT_2_	7 (20.0)	28 (80.0)	35	
pT_3_	9 (19.6)	37 (80.4)	46	
**pN status**				
pN_0_	35 (21.9)	125 (78.1)	160	0.319
pN_1_	5 (23.8)	16 (76.2)	21	
pN_2_-pN_3_	0(0.0)	8 (100.0)	8	
**TNM stage**				
I	22 (22.4)	76 (77.6)	98	0.800
II	6 (20.7)	23 (79.3)	29	
III	6 (21.4)	22 (78.6)	28	
IV	6 (17.6)	28 (82.4)	34	

*P values are obtained from χ^2^ test.

### Rap2a expression is increased in RCC cell lines

**Figure 2:**
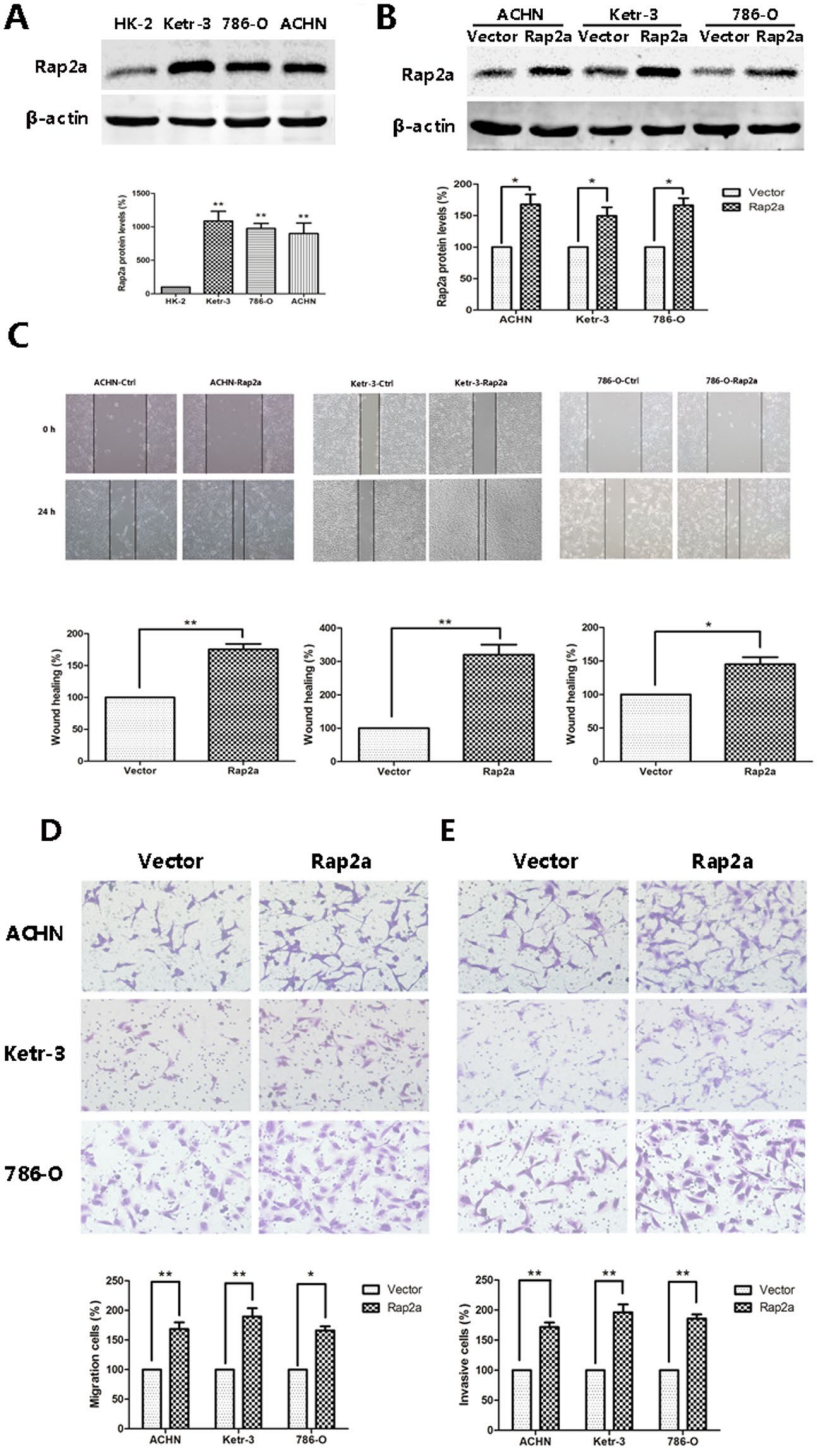
Effects of Rap2a overexpression on invasion and migration in RCC cells. (**A**) Western blot analysis of Rap2a expression in HK-2, Ketr-3, 786-O and ACHN. β-actin served as loading control. The intensity of Rap2a was quantified by densitometry (software: Image J, NIH). (**B**) ACHN, Ketr-3 and 786-O cell lines were transfected with Rap2a expressing or empty vector. Twenty-four hours post-transfection, Rap2a protein expression was detected by western blot. (**C**) Wound-healing assays were performed after Rap2a overexpression in ACHN, Ketr-3 and 786-O cells. (**D**,**E**) Cell migration was measured by using a migration assay following the transfection of RCC cells with Rap2a expression plasmid. Invasion assays were performed by using a similar procedure, except the polycarbonate filters was coated with Matrigel. Data are presented as mean ± SD (n = 3). *P < 0.05, **P < 0.01.

To investigate the expression of Rap2a in RCC development, western blot was used to examine the expression of Rap2a protein in 3 human RCC cell lines and normal renal tubular epithelial cell line HK-2. It was clear that the RCC cell lines had significant increased expression of Rap2a as compared with HK-2 (Fig. 2A). These results showed that Rap2a is up-regulated in RCC cell lines.

### Rap2a promotes RCC cell invasion and migration *in vitro*

The Rap2a overexpression plasmids were transfected into ACHN, Ketr-3 and 786-O cells and western blot showed that Rap2a was successfully transfected into RCC cells (Fig. 2B). To examine the oncogenic properties of Rap2a, we performed both wound healing and transwell assays. Our results showed that up-regulation of Rap2a strongly increased the wound closure in ACHN, Ketr-3 and 786-O cells (Fig. 2C). Moreover, transwell assays revealed a significant increase in the migration and invasion of Rap2a-overexpressing cells when compared with the control cells (Fig. 2D, E). These results indicated that Rap2a might have a significant effect on cell migration and invasion *in vitro*.

### Knockdown of Rap2a inhibits invasion and migration of RCC cell

**Figure 3:**
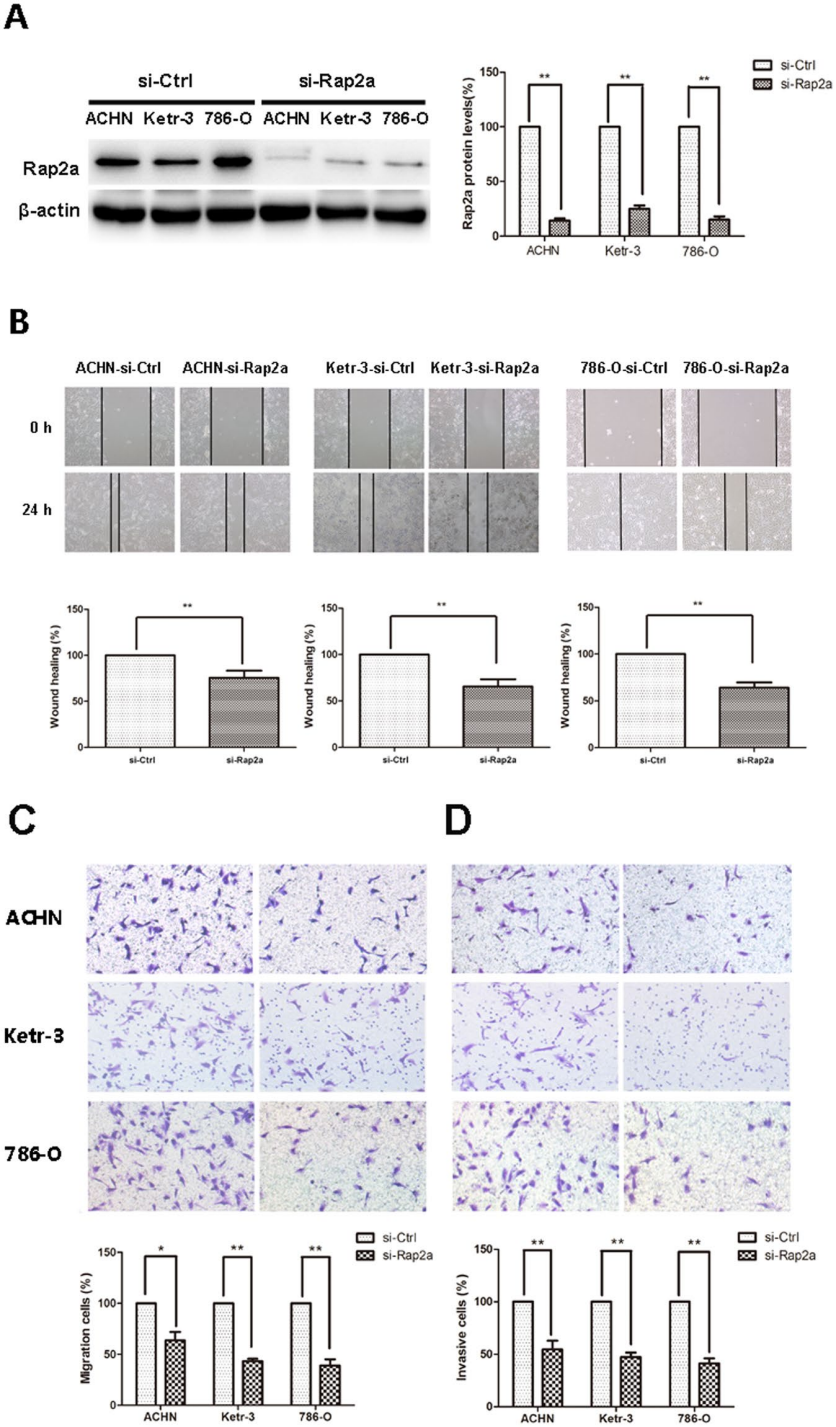
Effects of Rap2a knockdown on invasion and migration in RCC cells. (**A**) Western blot analysis of Rap2a knockdown in Ketr-3, 786-O, and ACHN group with Rap2a antibody. β-actin served as loading control. (**B**) Effects of Rap2a knockdown on the migration ability of RCC cells were examined by wound-healing assay. (**C**,**D**) Cell migration was measured by using a migration assay following the transfection of ACHN, Ketr-3 and 786-O cells with Rap2a siRNA. Invasion assays were performed by using a similar procedure. Data are presented as mean ± SD (n = 3). *P < 0.05, **P < 0.01.

To further investigate the effect of Rap2a on cell invasion and migration, we knocked down Rap2a in the RCC cell lines ACHN, Ketr-3 and 786-O by using small interfering RNA Rap2a. Western blot analysis revealed that siRap2a successfully knocked down Rap2a expression (Fig. 3A). Our results found that down-regulation of Rap2a decreased the wound closure and the migration ability of RCC cells (Fig. 3B,C). The similar result was got in cell invasion assay (Fig. 3D). These results demonstrated that knockdown of Rap2a inhibited invasion and migration of RCC cells *in vitro*.

### Rap2a has no effect on cell proliferation and apoptosis *in vitro*

**Figure 4:**
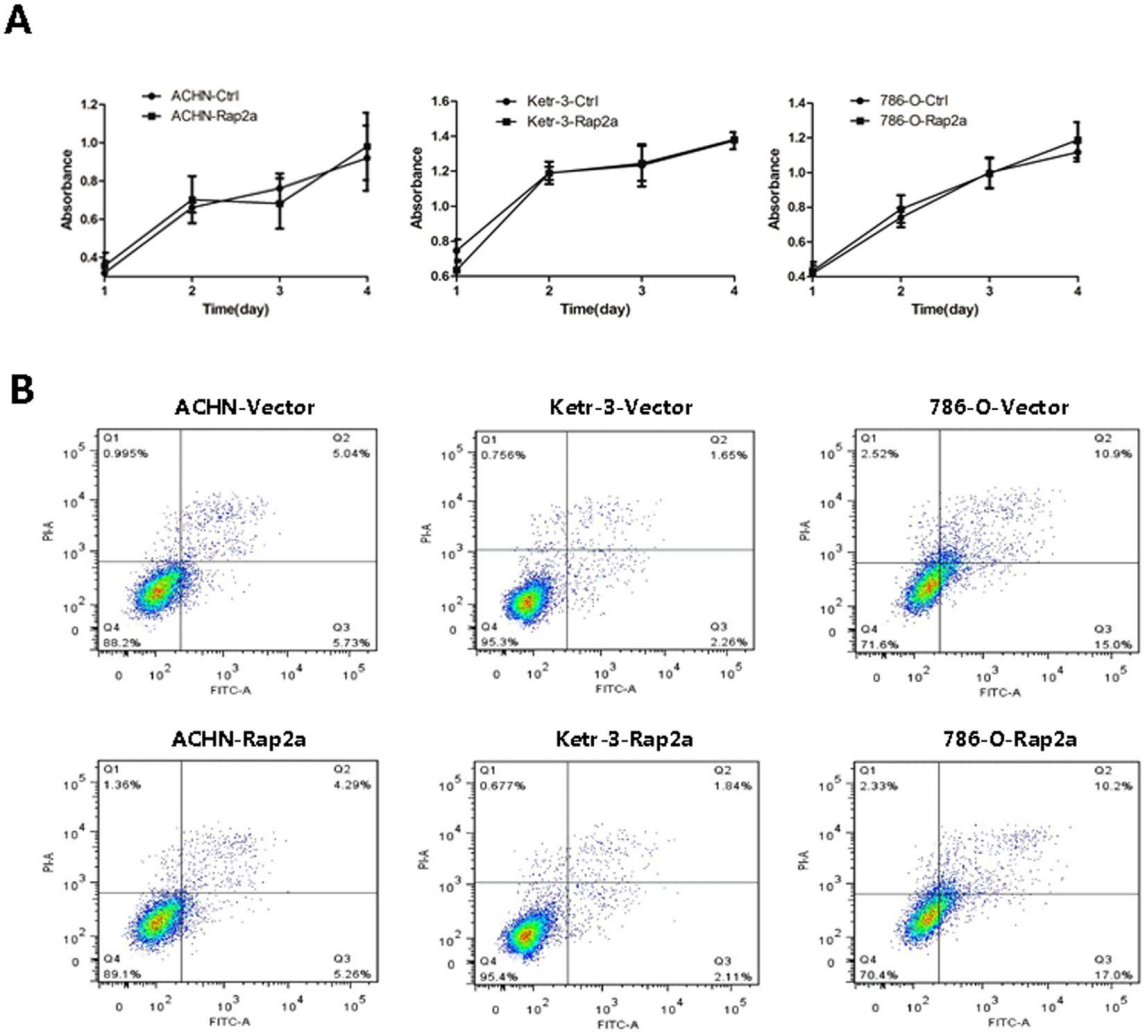
Effects of Rap2a overexpression on RCC cells proliferation. (**A**) CCK-8 assay was performed in ACHN, Ketr-3 and 786-O cells after transfection of pcDNA3.1-Rap2a/pcDNA3.1 empty vector expression plasmids for 24, 48, 72 and 96 h. (**B**) The apoptotic cells were determined by annexin V-FITC/PI staining using flow cytometry. Rap2a has no effect on the proliferation and apoptosis of RCC cells. Data are presented as mean ± SD (n = 3).

**Figure 5:**
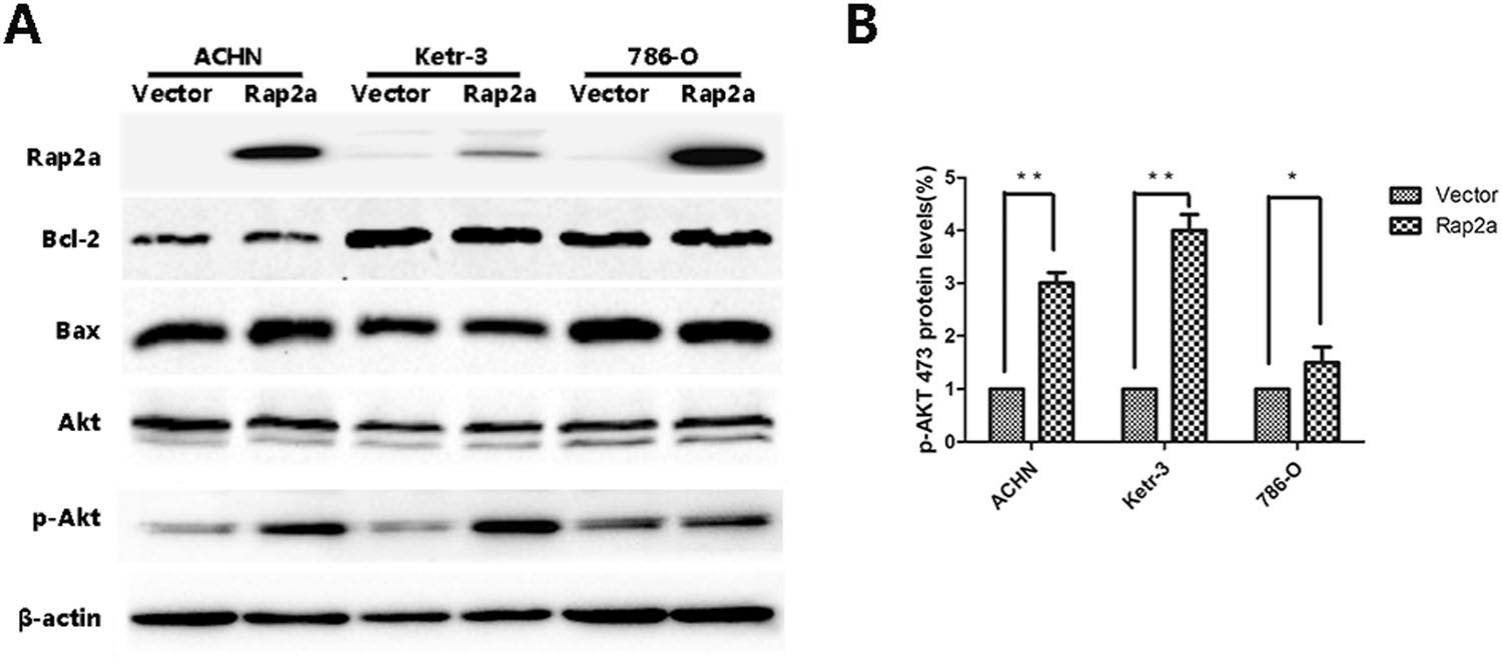
Akt phosphorylation involved in Rap2a-mediated migration and invasion of RCC cells. (**A**) Western blot analysis of the relative protein levels in RCC cells after Rap2a overexpression. The protein levels of Bcl-2, Bax and Akt had no significant difference between vector and Rap2a overexpression. However, p-Akt level was significantly increased after Rap2a overexpression. (**B**) Densitometric analysis of p-Akt473. The data are presented as mean ± SD from three independent experiments. *P < 0.05, **P < 0.01.

Based on the result from cell migration and invasion, we then investigated whether Rap2a affects carcinoma cell proliferation. After transfecting ACHN, Ketr-3 and 786-O with Rap2a overexpression plasmids, CCK-8 assay revealed that the cell proliferation had no significant difference between Rap2a-overexpression cells and the control cells (Fig. 4A). Consistent with previous result, no detectable difference of cell apoptosis were detected between two groups (Fig. 4B). In addition, the expression of Bcl-2 and Bax had no obvious change in Rap2a-overexpressed cells (Fig. 5A).

### Akt phosphorylation involved in Rap2a-mediated cancer cell migration and invasion

Occurrence of metastasis due to tumor development is the critical cause of most cancer-related death. Thus, we next explored the molecular pathways responsible for the metastasis effect of Rap2a. Tumor metastasis is a multistep process and can be affected by a variety of oncogene within the cancer cells. It is identified that Akt is a point of convergence for multiple oncogenic signaling pathways. In order to explore whether Rap2a regulates RCC cell migration and invasion by Akt, we detected the expression of Akt proteins. Our results showed that Akt expression did not change when Rap2a expression was up-regulated (Fig. 5A). However, Rap2a overexpression significantly increased the phosphorylation of Akt at serine 473 (p-Akt 473) (Fig. 5B). Thus, we inferred that Akt signaling pathway may be involved in Rap2a-mediated of RCC cells migration and invasion (Supplementary Fig. S1).

### Rap2a enhances RCC cells metastasis *in vivo*

**Figure 6:**
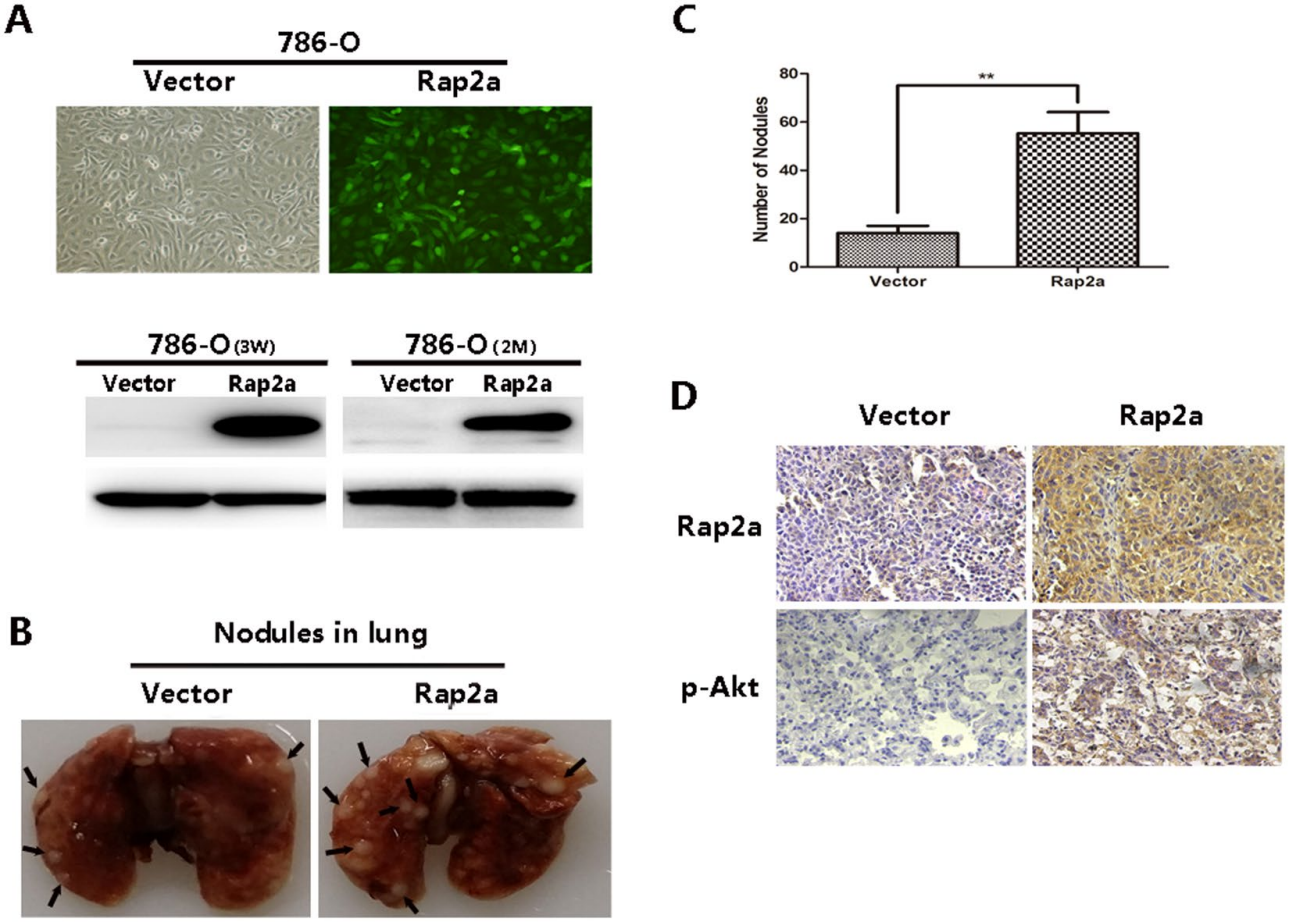
Rap2a enhances RCC cell metastasis *in vivo*. (**A**) 786-O cells were examined for their infection efficiency under the fluorescent microscope after lentivirus transfection (×100 magnification) (top panel). Western blot of Rap2a from Rap2a-overexpression 786-O cell lines and Ctrl-786-O cell lines selected with puromycin for 3 weeks after lentivirus infection. Rap2a expression levels remain stable without puromycin selection for 2 months (bottom panel). (**B**) Representative images of 10% buffered formalin fixed lungs with metastatic nodules 2 months after respective injection of Ctrl, Rap2a-overexpression 786-O cell lines. Arrows indicate metastatic nodules. The number of lung metastatic nodules was counted under a dissecting microscope. (**C**) A statistically dramatic increase in the number of the lung metastases was seen in Rap2a-overexpression group compared with the Ctrl group. Data are displayed with means ± SD from 10 mice in each group. (**D**) Immunostaining of Rap2a and p-Akt in metastatic nodules of Rap2a-overexpression and Ctrl 786-O groups. Rap2a and p-Akt expression in Rap2a-overexpression group were much higher compared with Ctrl group. **P < 0.01.

To further validate the implication of Rap2a in tumor migration and metastasis, Rap2a-overexpression-786-O cell lines was established by infecting with lentivirus packing Rap2a expression vector (Fig. 6A, left panel). Furthermore, Rap2a protein expression remained stable after 2 months without puromycin selection (Fig. 6A, right panel). So, we incubated them without adding puromycin for 2 months *in vitro*.

Rap2a-overexpression-786-O cells and Ctrl-786-O cells were respectively injected into two groups of nude mice tail vein. 2 months after injection, multiple tumor nodules were observed in the lungs of mice (Fig. 6B) and fixed in 10% buffered formalin for metastatic nodules counting and further histopathological analysis. The lungs in Rap2a group had more and larger detectable tumor nodules (Fig. 6B, right panel). A statistically dramatic increase in the number of the lung metastases was seen in Rap2a overexpression group compared with the control group (Fig. 6C). Immunohistochemical staining of metastatic nodules in lungs resected from nude mice confirmed that Rap2a overexpression resulted in much more markedly metastatic spread to the lungs of the mice compared to control vector. Moreover, Immunohistochemical staining showed that p-Akt expression in Rap2a overexpression group were much higher compared with control group. (Fig. 6D). These findings further validated our conclusion that Rap2a promoted cancer metastasis *in vivo*.

## Discussion

The Ras family of small GTPases is implicated in the mechanisms of a variety of cellular processes including cell adhesion, cell cycle control, differentiation, cytoskeletal organization and metabolic turnover^4, 12^. The previous studies have shown that Ras gene mutations or overexpression are involved in oncogenesis of various human tumors and have significantly worse survival than those without mutations^13, 14^. The Rap GTP-binding proteins, a subfamily of the Ras superfamily, control various biological processes via their downstream mitogen-activated protein kinases and PI3K signaling cascades^15^. Aberrant activation of Rap has received recognition for its role in cell cycle control, migration and invasion of cancer cells^16^. Rap2, a member of the Rap GTP-binding proteins, plays important roles in tumorigenesis and cancer progression^17, 18^. Emerging evidence suggests that high level of expression of Rap2a is involved in the invasion of follicular thyroid cancer^19^. Our previous studies have shown that Rap2a is involved in the cell migration and invasion of lung cancer cells^11^. In this study, our results demonstrated that Rap2a expression is predominantly increased in RCC cell lines, which is consistent with the result of our previous study indicating that the levels of Rap2a expression are all significantly higher in tumor tissues than those in adjacent, normal renal tissues. Taken together, we infer that Rap2a might play an important role in the development of human RCC.

RCC is an immunogenic and proangiogenic cancer^20^. It accounts for 3% of adult malignancies and 90% of neoplasms stemming from the kidney^21^. Approximately 20–40% of patients with RCC will die of metastatic disease, because metastases are often present at diagnosis and relapse following nephrectomy. Patients with metastatic disease have a poor prognosis, with 5-year survival rates being less than 10%^22^. Tumor metastasis is a multiple process that can be regulated by various genes. Cell migration and invasion are considered to be critical factors for malignant tumor metastasis. The management of metastatic renal cell carcinoma in clinical is always a difficult problem to solve. Although antivascular endothelial growth factor (VEGF) therapies achieve impressive responses in some patients, many tumors eventually develop resistance to such therapy^20^. In recent years, molecular targeted therapies and the biological predictive markers able to identify patients have dramatically improved the prognosis of patients with metastatic RCC^23–25^. As increasing evidence of Rap2a overexpression in tumor progression had been discovered, the mechanisms underlying the regulations attracted notable attention. It is important to assess whether Rap2a gene can be used as biomarkers to predict tumor metastasis and guide the choice of therapy, which is why we investigated the level of Rap2a expression in RCC patients as well as their associations with clinicopatological features.

In the present study, we used immunohistochemical technique, cell proliferation assay, wound-healing assay, migration assay, invasion assay and western blot to investigate the role of Rap2a in RCC cells. We first identified increased expression of Rap2a protein in RCC tissues. We found that Rap2a expression was significantly increased in RCC tissues compared with tumor adjacent normal renal tissues. These observations suggest that Rap2a is an important prognostic factor in RCC. However, we did not find significant correlation between Rap2a expression with TNM stages or other clinicopathologic variables, including age, gender and tumor size. Tumor cell migration and invasion are essential steps in the process of metastasis. Our results demonstrated that the upregulation of Rap2a promoted the migratory and invasive capacities of cancer cells. Meanwhile, the downregulation of Rap2a inhibited the migratory and invasive capacities of cancer cells. CCK-8 and Annexin V/PI assays showed that Rap2a had no effect on the proliferation and apoptosis of cancer cells. Consistent with this result, cancer cells treated with Rap2a did not show any changes in the expression levels of Bax and Bcl-2. These findings suggested that Rap2a could promote cancer cell metastasis instead of proliferation. Furthermore, our studies showed that Rap2a regulate the phosphorylation level of Akt. To the best of our knowledge, this is the first study demonstrating that Rap2a might play some potential roles in the development and progression of RCC cells. The phosphorylation level of Akt at Ser 473 is commonly associated with tumor invasion and metastasis and p-Akt has been considered as biomarkers in many types and stages of cancer^26^. The present results showed that the overexpression of Rap2a enhances the protein levels of p-Akt. Thus, Rap2a enhances migration and invasion of RCC cells and that this effect may be mediated by increased p-Akt expression. These findings indicate the involvement of p-Akt in Rap2a-induced invasion of tumor cells.

Here, we investigated the role of Rap2a in RCC carcinogenesis *in vivo*. Our results showed that Rap2a overexpression in RCC cells significantly enhanced the formation of metastasis nodules in lung of nude mice. Moreover, trends of p-Akt immunostaining in metastasis nodules of Rap2a overexpression groups were coincident with *in vitro* experiments. Our *in vivo* experiment about Rap2a provided more evidence to support the contribution of Rap2a overexpression in tumor development.

In general, this study provides evidence that Rap2a was an independent prognostic factor of worse outcome in RCC patients. The overexpression of Rap2a may contribute to the promotion of tumor invasion and migration and has no effect on the proliferation and apoptosis of RCC cells. In contrast, downregulation of Rap2a inhibits the migration and invasive ability of cancer cells. Furthermore, we demonstrated that Rap2a could enhance the phosphorylation level of Akt *in vitro*. Thus, elevated Akt phosphorylation may participate in Rap2a mediated cancer cell migration and invasion. Our study firstly provided the *in vitro* and *in vivo* evidences that targeting Rap2a might constitute a potential treatment modality for RCC and represent a new therapy to suppress RCC metastasis.

## Electronic supplementary material

supplementary information

## References

[1] Linehan WM, Rathmell WK (2012). Kidney cancer. Urol oncol..

[2] Linehan WM, Srinivasan R, Schmidt LS (2010). The genetic basis of kidney cancer: a metabolic disease. Nat Rev Urol..

[3] Ball MW, Allaf ME, Drake CG (2016). Recent advances in immunotherapy for kidney cancer. Discov Med..

[4] Mittal V, Linder ME (2006). Biochemical characterization of RGS14: RGS14 activity towards G-protein alpha subunits is independent of its binding to Rap2A. Biochem J..

[5] Paganini S (2006). Identification and biochemical characterization of Rap2C, a new member of the Rap family of small GTP-binding proteins. Biochimie..

[6] Albright CF, Giddings BW, Liu J, Vito M, Weinberg RA (1993). Characterization of a guanine nucleotide dissociation stimulator for a ras-related GTPase. EMBO J..

[7] Bokoch GM (1993). Biology of the Rap proteins, members of the ras superfamily of GTP-binding proteins. Biochem J..

[8] Pasheva E, Janoueix-Lerosey I, Tavitian A, de, Gunzburg J (1994). Characterization of the Ras-related RAP2A protein expressed in the baculovirus-insect cell system: processing of the protein in insect cells and comparison with the bacterially produced unprocessed form. Biochem Biophys Res Commun..

[9] Wu J, Sang M, Cao W, Zheng J, Pei D (2014). Identification analysis of eukaryotic expression plasmid Rap2a and its effect on the migration of lung cancer cells. Zhongguo Fei Ai Za Zhi..

[10] Lee YE (2015). The prognostic impact of RAP2A expression in patients with early and locoregionally advanced nasopharyngeal carcinoma in an endemic area. Am J Transl Res..

[11] Wu JX, Zhang DG, Zheng JN, Pei DS (2015). Rap2a is a novel target gene of p53 and regulates cancer cell migration and invasion. Cell Signal..

[12] Rebhun JF, Chen H, Quilliam LA (2000). Identification and characterization of a new family of guanine nucleotide exchange factors for the ras-related GTPase Ral. J Biol Chem..

[13] Rodenhuis S, Slebos RJ (1990). The ras oncogenes in human lung cancer. Am Rev Respir Dis..

[14] Mascaux C (2005). The role of RAS oncogene in survival of patients with lung cancer: a systematic review of the literature with meta-analysis. Br J Cancer..

[15] Stornetta RL, Zhu JJ (2011). Ras and Rap signaling in synaptic plasticity and mental disorders. Neuroscientist..

[16] Bailey CL, Kelly P, Casey PJ (2009). Activation of Rap1 promotes prostate cancer metastasis. Cancer Res..

[17] Taira K (2004). The Traf2- and Nck-interacting kinase as a putative effector of Rap2 to regulate actin cytoskeleton. J Biol Chem..

[18] Bigler D, Gioeli D, Conaway MR, Weber MJ, Theodorescu D (2007). Rap2 regulates androgen sensitivity in human prostate cancer cells. Prostate..

[19] Prabakaran I, Grau JR, Lewis R, Fraker DL, Guvakova MA (2011). Rap2A Is Upregulated in Invasive Cells Dissected from Follicular Thyroid Cancer. J Thyroid Res..

[20] Fukuda T (2016). Higher preoperative serum levels of PD-L1 and B7-H4 are associated with invasive and metastatic potential and predictable for poor response to VEGF-targeted therapy and unfavorable prognosis of renal cell carcinoma. Cancer Med..

[21] Chow WH, Devesa SS, Warren JL, Fraumeni JF (1999). Rising incidence of renal cell cancer in the United States. JAMA..

[22] Ueda K (2016). Long-term response of over ten years with sorafenib monotherapy in metastatic renal cell carcinoma: a case report. J Med Case Rep..

[23] Escudier B (2007). Sorafenib in advanced clear-cell renal-cell carcinoma. N Engl J Med..

[24] Hudes G (2007). Temsirolimus, interferon alfa, or both for advanced renal-cell carcinoma. N Engl J Med..

[25] Motzer RJ (2007). Sunitinib versus interferon alfa in metastatic renal-cell carcinoma. N Engl J Med.

[26] Yin S (2016). Wip1 suppresses ovarian cancer metastasis through the ATM/Akt/Snail mediated signaling. Oncotarget..

