# Author Correction: Rap2a serves as a potential prognostic indicator of renal cell carcinoma and promotes its migration and invasion through up-regulating p-Akt

**DOI:** 10.1038/s41598-021-93823-3

**Published:** 2021-07-15

**Authors:** Jin-Xia Wu, Wen-Qi Du, Xiu-Cun Wang, Lu-Lu Wei, Fu-Chun Huo, Yao-Jie Pan, Xiao-Jin Wu, Dong-Sheng Pei

**Affiliations:** 1 Department of Pathology, Xuzhou Medical University, Xuzhou, 221004 China; 2 Department of Physiology, Xuzhou Medical University, Xuzhou, 221004 China; 3 Jiangsu Key Laboratory of Biological Cancer Therapy, Xuzhou Medical University, Xuzhou, 221002 China; 4 Jiangsu Center for the Collaboration and Innovation of Cancer Biotherapy, Xuzhou Medical University, Xuzhou, 221002 China; 5 Department of Neurosurgery, The Affiliated Hospital of Xuzhou Medical University, Xuzhou, 221002 China; 6 Department of Radiation Oncology, The First People’s Hospital of Xuzhou, Xuzhou, 221002 China

Correction to: *Scientific Reports* 10.1038/s41598-017-06162-7, published online 26 July 2017

The original version of this Article contained errors in Figure 2, where the incorrect images were used in the Ketr-3/Rap2a panel of Figure 2D, and the Ketr-3/Vector panel of Figure 2E.

The original Figure 2 and accompanying legend appears below.

**Figure 2:**
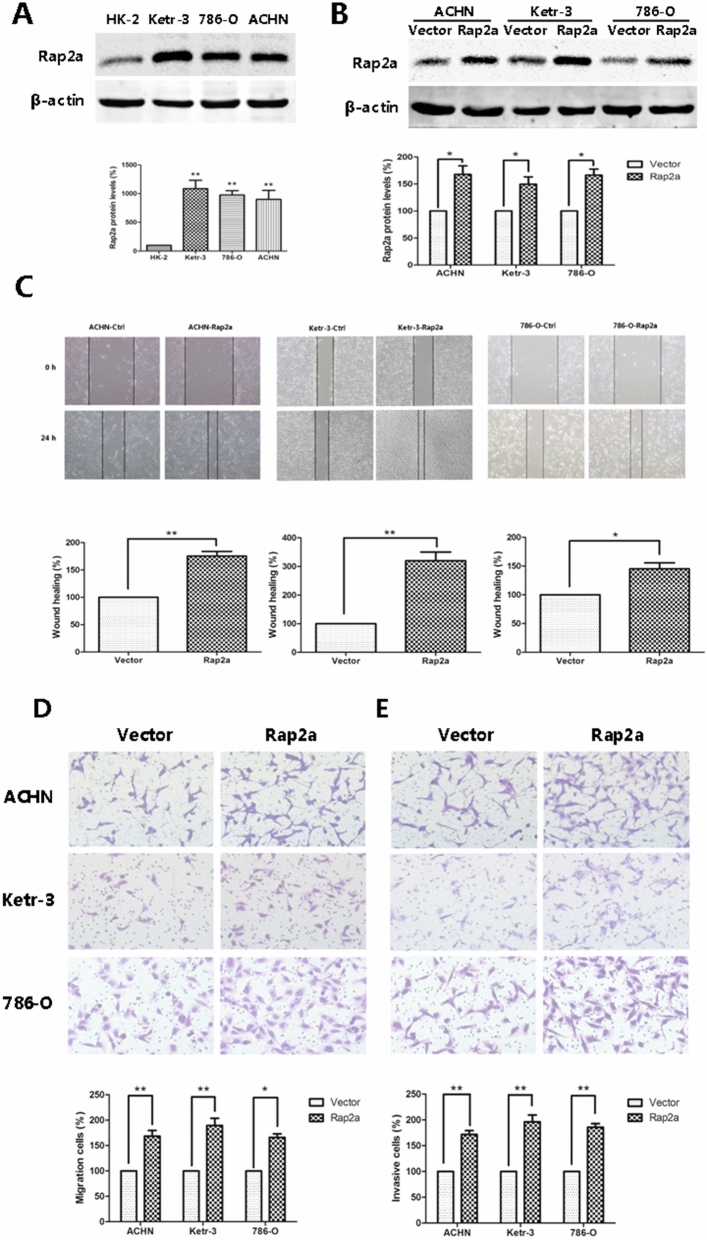
Effects of Rap2a overexpression on invasion and migration in RCC cells. (**A**) Western blot analysis of Rap2a expression in HK-2, Ketr-3, 786-O and ACHN. β-actin served as loading control. The intensity of Rap2a was quantified by densitometry (software: Image J, NIH). (**B**) ACHN, Ketr-3 and 786-O cell lines were transfected with Rap2a expressing or empty vector. Twenty-four hours post-transfection, Rap2a protein expression was detected by western blot. (**C**) Wound-healing assays were performed after Rap2a overexpression in ACHN, Ketr-3 and 786-O cells. (**D**,**E**) Cell migration was measured by using a migration assay following the transfection of RCC cells with Rap2a expression plasmid. Invasion assays were performed by using a similar procedure, except the polycarbonate filters was coated with Matrigel. Data are presented as mean ± SD (n = 3). *P < 0.05, **P < 0.01.

The original Article has been corrected.

