# Supplementary material for: Rap2a serves as a potential prognostic indicator of renal cell carcinoma and promotes its migration and invasion through up-regulating p-Akt
Source: Sci Rep. 2017 Jul 26;7:6623. doi: 10.1038/s41598-017-06162-7 (PMC5529368; doi:10.1038/s41598-017-06162-7)
Supplement: Supplementary file 1 — supplementary information [file 41598_2017_6162_MOESM1_ESM.doc]

**Rap2a serves as a potential prognostic indicator of renal cell carcinoma and promotes its migration and invasion through up-regulating p-Akt**

Authors: Jin-Xia Wu^2*^, Wen-Qi Du^3*^, Xiu-Cun Wang^5*^, Lu-Lu Wei^1^, Fu-Chun Huo^3^, Yao-Jie Pan^3^, Xiao-Jin Wu^6^ & Dong-Sheng Pei^1,3,4^

Affiliation:

1 Department of Pathology, Xuzhou Medical University, Xuzhou 221004, China

2 Department of Physiology, Xuzhou Medical University, Xuzhou 221004, China

3 Jiangsu Key Laboratory of Biological Cancer Therapy, Xuzhou Medical University, Xuzhou 221002, China

4 Jiangsu Center for the Collaboration and Innovation of Cancer Biotherapy, Xuzhou Medical University, Xuzhou 221002, China

5 Department of Neurosurgery, The Affiliated Hospital of Xuzhou Medical University, Xuzhou 221002, China

6 Department of Radiation Oncology, The First People’s Hospital of Xuzhou, Xuzhou 221002, China

Supplementary Fig. S1: Western blot analysis of the relative protein levels of TIMP2, E-cadherin, Vimentin, FAK, NF-kB and p-ERK after Rap2a overexpression for ACHN, Ketr-3 and 786-O cell lines.


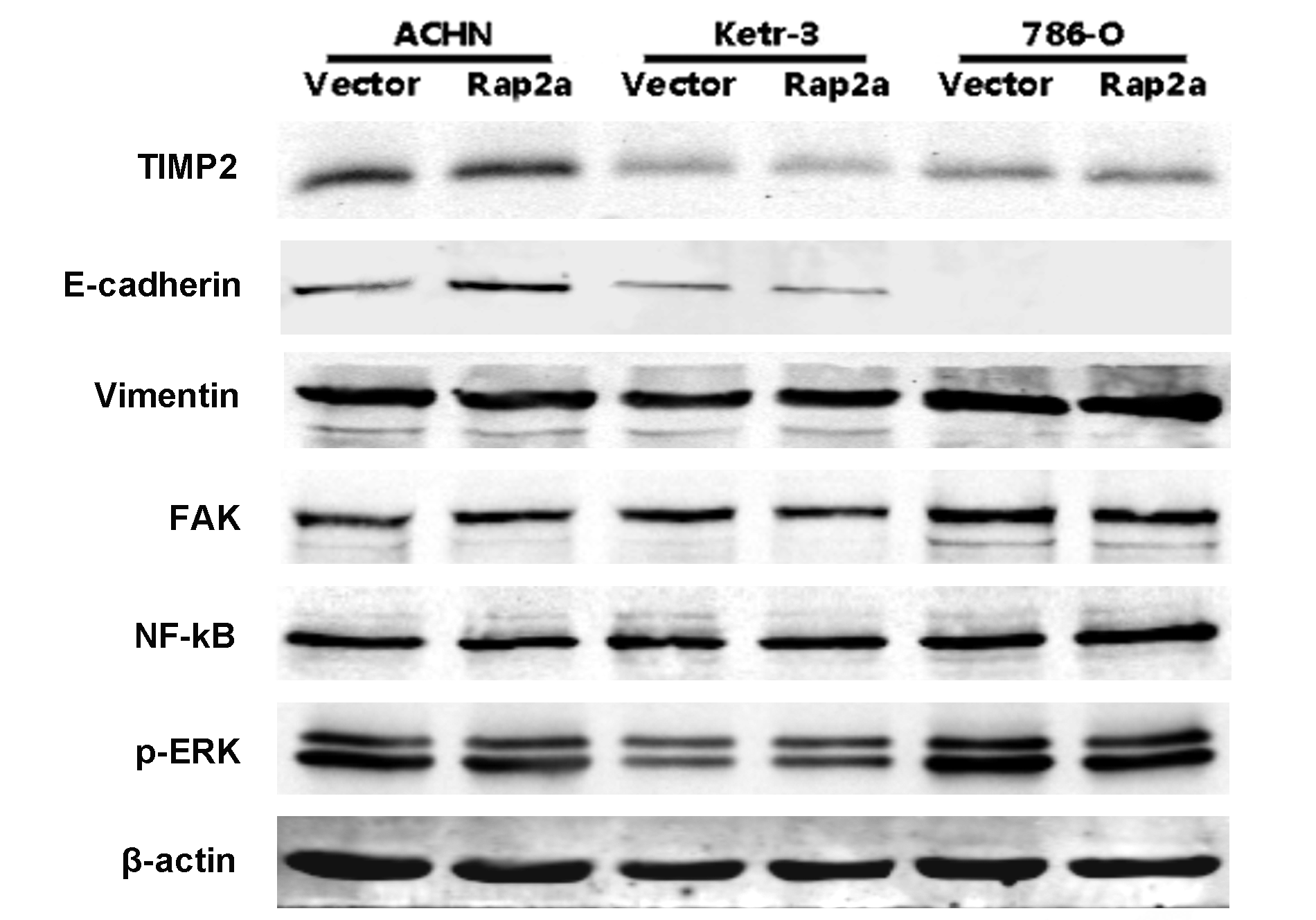


Unprocessed original scans for all of the blots:
Image acquisition tools:

(1) Odyssey Two-Color Infrared Imaging System (LI-COR Biotechnology, Lincoln, Nebraska, USA);

(2) Tanon Chemiluminescence imaging analysis system (shanghai, China)

Fig.2

A

Marker HK-2 Ketr-3 786-O ACHN


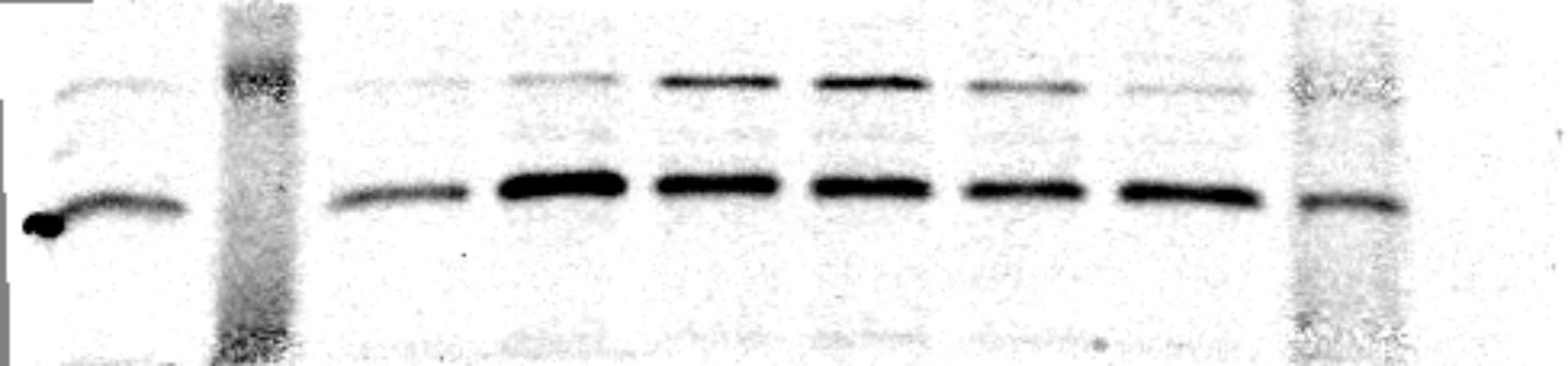


25

15


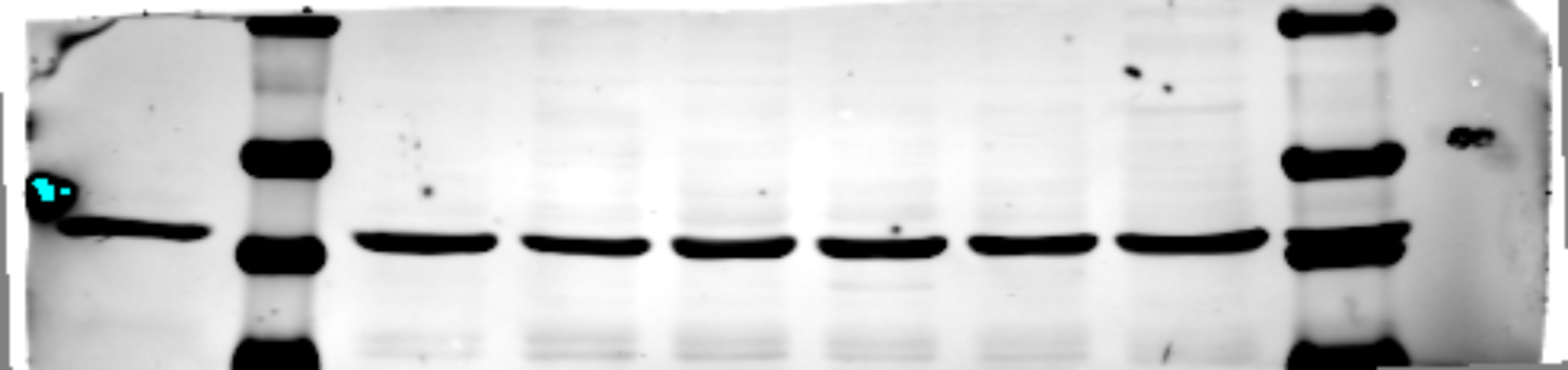


70

55

40

35

Rap2a (21KD)

β-actin

(43KD)

B

Rap2a (21KD)

β-actin

(43KD)


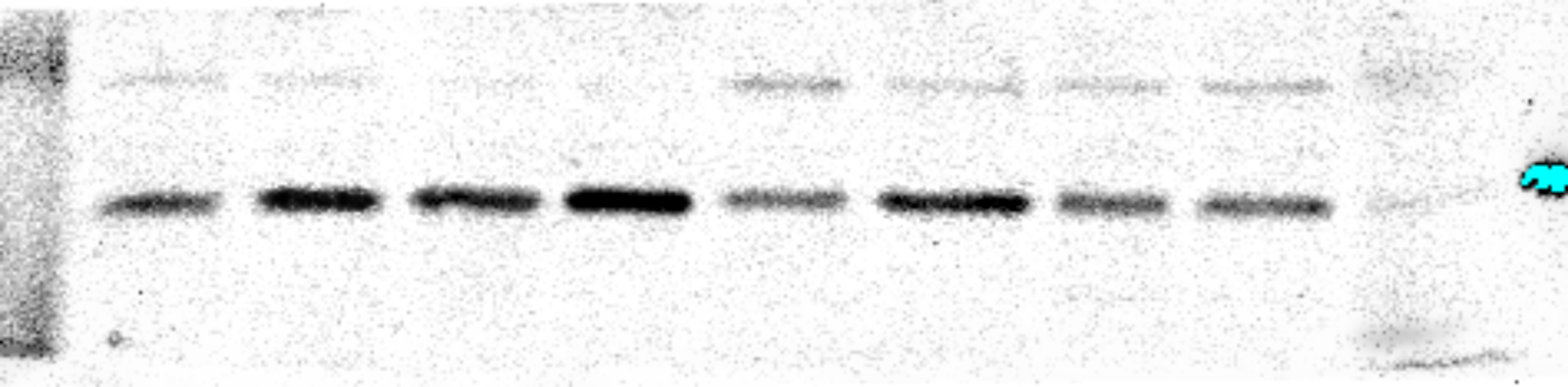


25

15


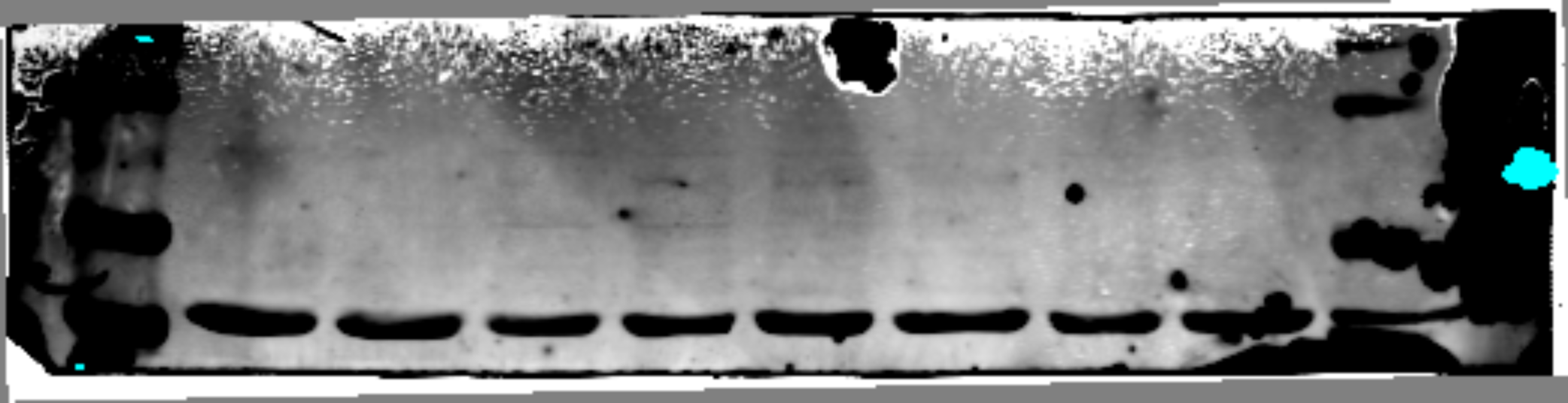


70

55

40

Marker Vector Rap2a Vector Rap2a Vector Rap2a

ACHN Ketr-3 786-O

Fig.3

Rap2a (21KD)

β-actin

(43KD)


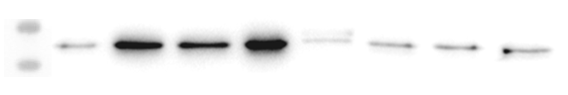

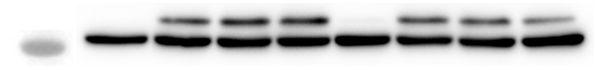


15

25

40

Marker ACHN Ketr-3 786-O ACHN Ketr-3 786-O

si-Ctrl si-Rap2a

A

Fig.5

A

Marker Vector Rap2a Vector Rap2a Vector Rap2a

ACHN Ketr-3 786-O


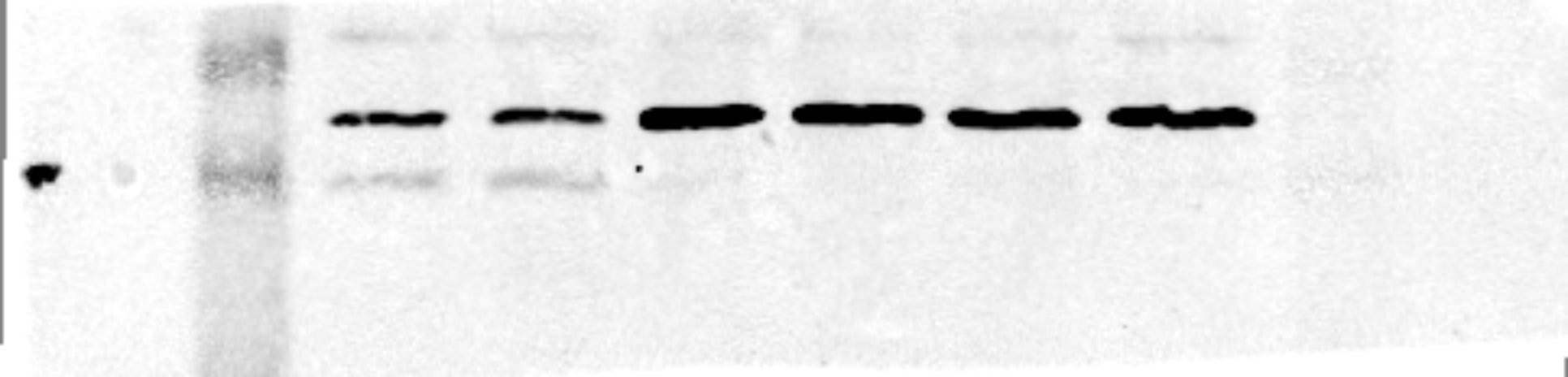


35

25


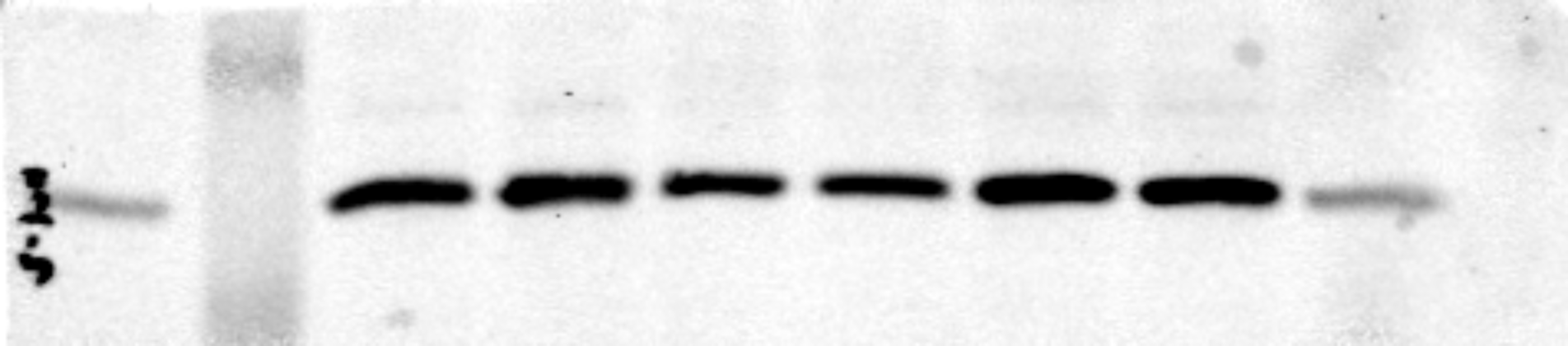


25

15

70

55

40


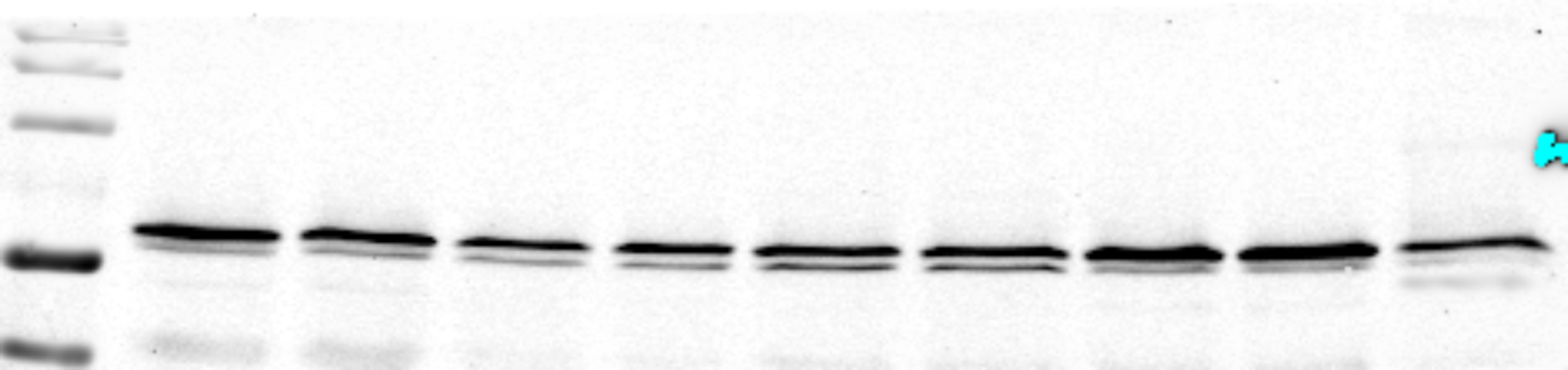

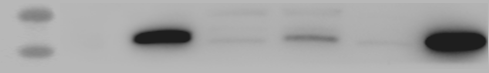


25

15


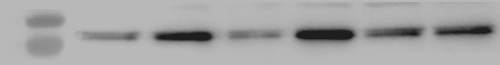


70

55


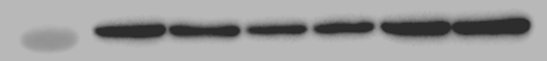


40

Rap2a

(21KD)

Bcl-2 (26KD)

Bax

(23KD)

Akt

(60KD)

p-Akt

(60KD)

β-actin

(43KD)

Fig.6

A

786-O (2M)


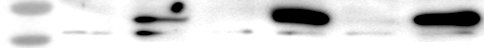

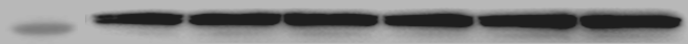


15

25

40

Vector Rap2a

Rap2a

(21KD)

β-actin

(43KD)
